# Theory-based and evidence-based nursing interventions for the prevention of ICU-acquired weakness in the intensive care unit: A systematic review

**DOI:** 10.1371/journal.pone.0308291

**Published:** 2024-09-13

**Authors:** Qin Xu, Jie Tan, Yixuan Wang, Manli Tang

**Affiliations:** Department of Nursing, Tongji Hospital, Tongji Medical College, Huazhong University of Science and Technology, Wuhan, China; University of Toronto, CANADA

## Abstract

**Objectives:**

To synthesise and map the evidence of a theory- and evidence-based nursing intervention for the prevention of ICU-acquired weakness and evaluate its effectiveness in terms of the incidence of ICU-acquired weakness, incidence of delirium, and length of hospital stay.

**Methods:**

We searched PubMed, CINAHL, MEDLINE, Academic Search Complete, Embase, Scopus, Web of Science and the Cochrane Library from database inception to November 2023. The eligible studies focused on critically ill patients in the intensive care unit, used a theory- and evidence-based nursing intervention, and reported the incidence of ICU-acquired weakness and/or used the Medical Research Council Scale. The methodological quality of the included studies was critically appraised by two authors using the appropriate Joanna Briggs Institute appraisal tool for randomised controlled trials, quasi-experimental studies, and cohort studies. Additionally, the weighted kappa coefficient was used to assess inter-rater agreement of the quality assessment. Data were reported using a narrative synthesis. This systematic review was registered by the International Prospective Register of Systematic Review (PROSPERO; CRD42023477011).

**Results:**

A total of 5162 studies were initially retrieved, and 9 studies were eventually included after screening. This systematic review revealed that preventive nursing interventions for ICU-acquired weakness mainly include (a) physiotherapy, including neuromuscular electrical stimulation and early rehabilitation, and (b) nutritional support. In addition, (c) airway management, (d) sedation and analgesia management, (e) complication prevention (delirium, stress injury and deep vein thrombosis prevention), and (f) psychological care were also provided. The theories are dominated by goal-oriented theories, and the evidence is mainly the ABCDE bundle in the included studies. The results show that theory- or evidence-based nursing interventions are effective in reducing the incidence of ICU-acquired weakness (or improving the Medical Research Council Scale scores), decreasing the incidence of delirium, shortening the length of hospital stay, and improving patients’ self-care and quality of life.

**Conclusion:**

Theory- and evidence-based nursing interventions have good results in preventing ICU-acquired weakness in critically ill patients. Current nursing interventions favour a combination of multiple interventions rather than just a single intervention. Therefore, preventive measures for ICU-acquired weakness should be viewed as complex interventions and should be based on theory or evidence. This systematic review is based on a small number of trials. Thus, more high-quality randomised controlled trials are needed to draw definitive conclusions about the impact of theory- and evidence-based nursing interventions on the prevention of ICU-acquired weakness.

## Introduction

ICU-acquired weakness (ICU-AW) is the most common neuromuscular injury affecting disease progression and prognosis in the intensive care unit (ICU) [1, 2]. ICU-AW is a syndrome of generalised limb weakness that occurs without a clear cause in critically ill patients and is characterised by mild paralysis or tetraplegia, decreased reflexes, myasthenia gravis and weaning failure [3]. The subtypes of ICU-AW include critical illness polyneuropathy, critical illness myopathy and critical illness neuromyopathy [2, 3]. ICU-AW usually occurs symmetrically, with the limb muscles and respiratory muscles being more involved than the facial muscles [4]. Most of the diaphragm is also involved, leading to prolonged mechanical ventilation and difficulty in extrication [4]. Currently, the Medical Research Council Scale (MRC) is widely recognised as an important tool for the diagnosis of ICU-AW, with an MRC score of less than 48 being considered ICU-AW.

ICU patients are characterised by severe illness, a complex and prolonged treatment course, and multiple ICU-AW risk factors (e.g., prolonged bed rest, duration of mechanical ventilation, use of sedative drugs and corticosteroids, hyperglycaemia and parenteral nutrition) [2, 5]. The prevalence of ICU-AW in ICU patients is approximately 43% [6] and can be as high as 36% after discharge [7]. In propensity score matching, the risk of death at 1 and 5 years after ICU discharge depends on the persistence and severity of muscle weakness at the time of discharge, and patients with a more severe degree of persistent muscle weakness (MRC<35) are more likely to die later in life [8]. The occurrence of ICU-AW prolongs the duration of ventilator-assisted ventilation and increases the length of stay in the ICU and the mortality rate of patients. Moreover, patients may experience limb dysfunction after discharge from the hospital, which seriously affects their prognosis and quality of life [9]. In addition, ICU-AW increases the degree of psychological stress and financial burden on patients [2].

Currently, the pathogenesis of ICU-AW is uncertain, and there are no specific drugs or targeted therapies available [4]. Therefore, early identification and early intervention for ICU-AW are extremely important for critically ill patients. Some scholars have used risk prediction models to identify populations at high risk for ICU-AW [5]. Nursing interventions play an important role in the early prevention of ICU-AW. Nursing interventions are nurse-led actions taken to help patients heal and recover from illness and injury [10]. Common nursing interventions for the prevention of ICU-AW include early rehabilitation and nutritional support [11–13]. When assessing early rehabilitation interventions, the content and timing of interventions varies across studies due to the diversity of interventions (e.g., functional limb exercises, joint mobility training, and bed cycling/walking). Morris et al. classified early rehabilitation for critically ill patients into four levels based on the patient’s level of consciousness and muscle strength [14]. Hodgson et al. proposed a five-level early activity programme based on goal-directed theory, which determines a patient’s activity level using the ICU Mobility Scale score and sets matching activity goals [15]. Therefore, there is a greater need for relevant theories to guide nursing interventions.

There have been an increasing number of clinical trials examining theory- and evidence-based interventions for the prevention of ICU-AW. However, no study has yet conducted a relevant systematic review or meta-analysis. Thus, this systematic review provides a holistic perspective on the management of ICU-AW by systematically mapping and summarising theory- and evidence-based nursing interventions for the prevention of ICU-AW in critically ill patients and helps to bridge current evidence and clinical practice.

## Methods

### Design

This systematic review was designed according to the guidelines of the Preferred Reporting Items for Systematic Reviews and Meta-analyses Protocols (PRISMA) checklist [16]. The data were analysed narratively and synthesised using the Synthesis Without Meta-analysis (SWiM) guidelines [17]. The PICOS acronym was used in this study as follows:

PICOS

- P: Critically ill patients in the ICU.
- I: Theory-based and evidence-based nursing interventions.
- C: Routine care/other intervention.
- O: Primary—The incidence of ICU-AW (a total MRC score below 48) and the MRC score.

Secondary—The incidence of delirium (assessed using the Confusion Assessment Method for the ICU), duration of mechanical ventilation, duration of ICU stay and length of hospital stay.

- S: Randomised controlled trials, cohort studies and quasi experimental studies.

### Protocol and registration

This systematic review was registered by the International Prospective Register of Systematic Review (PROSPERO; CRD42023477011).

### Search methods

A comprehensive literature search was conducted using PubMed, EBSCOhost (CINAHL, MEDLINE and Academic Search Complete), Embase, Scopus, Web of Science, and the Cochrane Library from database inception to 1^st^ November 2023. The search terms included intensive care unit-acquired weakness (and related terms), and intensive care unit (and related terms). There were no restrictions imposed on the language or publication status. The specific search strategies and results for all the searched databases are detailed in Supporting information.

### Inclusion and exclusion criteria

Studies were eligible for inclusion if they met the following criteria: (1) the population included adult patients in the ICU (≥18 years old or according to local law), (2) outcomes included the incidence of ICU-AW and/or MRC sum scores, and (3) nursing interventions were based on theory or evidence. The exclusion criteria for studies were as follows: (1) study interventions were performed after ICU discharge, (2) the study was published as a review, meta-analysis, or case report, or (3) the study was a piece of grey literature such as a dissertation, letter to the editor, committee report, government report or conference paper.

### Study selection

EndNote© 20.6 software was used to manage and screen all articles. All of the retrieved articles were imported into EndNote. Duplicate studies were identified and removed by the software and manually. Two independent reviewers (QX; YW) read the title, keywords and abstract of the screened literature to ensure compliance with the inclusion criteria. Articles that met the inclusion criteria were carefully read in full and then evaluated by two independent reviewers (QX; YW). The two researchers discussed and then decided whether the articles were eligible for inclusion. Disagreements were resolved through discussion with a third reviewer (JT).

### Quality appraisal

Two independent reviewers (QX; YW) assessed the quality of the included studies. The inter-rater agreement between the two researchers in the quality appraisal was calculated by Cohen’s kappa. For controversial studies, the quality was assessed after discussion with a third reviewer (JT). The Joanna Briggs Institute (JBI) critical appraisal tools for randomised controlled trials, quasi-experimental studies, and cohort studies were used to evaluate the quality of the included studies.

### Data extraction

Data extraction was performed by two independent reviewers (QX; YW). A summary table was created to include the first author, year of publication, sample size, demographics, nursing intervention and clinical outcomes from each study (Tables 1 and 2).

**Table 1:** Characteristics of the included studies.

Authors	Country	Methods	Setting and disease	Samples/Age	Gender	Quality score /Total score
Bian et al. (2019)	China	Quasi-experimental study	ICU (Multiple trauma severe pneumonia, severe pancreatitis, respiratory failure et al.)	Total number of patients 96 Intervention group = 48/ (65.17 ± 12.12) Control group = 48/ (68.98 ± 10.86)	F = 26 M = 70	8/9
Hodgson et al. (2016)	Australia	Randomized controlled trial	Five ICUs in Australia and New Zealand	Total number of patients 50 Intervention group = 29/ (64 ± 12) Control group = 21/ (53 ± 15)	F = 20 M = 30	11/13
Lin et al. (2023)	China	Randomized controlled trial	SICU (Acute type A aortic dissection)	Total number of patients 77 Intervention group = 39/ (53.44 ± 12.06) Control group = 38/ (52.32 ± 13.86)	F = 18 M = 59	10/13
Yu et al. (2021)	China	Quasi-experimental study	Two wards in ICU (Diseases of digestive, respiratory, neurological et al.)	Total number of patients 74 Intervention group = 38/ (68.34 ± 5.77) Control group = 36/ (68.89 ± 3.68)	F = 30 M = 44	8/9
Zhang et al. (2023)	China	Quasi-experimental study	ICU	Total number of patients 120 Intervention group = 60/ (61.54 ± 17.06) Control group = 60/ (61.26 ± 15.85)	F = 56 M = 64	8/9
Frade-Mera et al. (2022)	Spain	Cohort study	80 ICUs (Heart surgery, trauma, neurosurgery and other surgery et al.)	Total number of patients 605 ◾ Analgosedation algorithms protocol: Intervention group = 133/ (62 [50, 72]) Control group = 472/ (67 [55, 75]) ◾ Delirium prevention and management protocol: Intervention group = 68/ (69 [52, 76]) Control group = 534/ (65 [54, 74]) ◾ Early mobilization protocol: Intervention group = 51/ (67 [53, 77]) Control group = 554/ (65 [54, 74])	F = 182 M = 423	10/11
Zhao et al. (2022)	China	Randomized controlled trial	ICU (Myocardial infarction)	Total number of patients 227 Intervention group = 115/ (55.7 ± 3.28) Control group = 112/ (57.3 ± 5.53)	F = 97 M = 130	8/13
Ying W et al. (2021)	China	Randomized controlled trial	One RICU	Total number of patients 72 Intervention group = 36/ (47.17 ± 11.63) Control group = 36/ (46.76 ± 13.36)	F = 31 M = 41	9/13
Han et al. (2020)	China	Randomized controlled trial	ICU (Lung infection, COPD, fulminant myocarditis, polytrauma, severe pancreatitis et al.)	Total number of patients 70 Intervention group = 35/ (56.57 ± 16.82) Control group = 35/ (52.57 ± 12.97)	F = 27 M = 43	8/13

**Table 2:** Description of the intervention, measurement and results.

Authors	Theory and evidence of nursing	Intervention description	Outcomes
Bian et al. (2019) China	Theory-based: eCASH concept	1/ Patient-centred care without excessive sedation: ● Early comfort using analgesia: Remifentanil was used for analgesia on prescription, with a maintenance dose of 0.1 to 0.3 μg/(kg·h) after loading. The pain assessment tools were selected according to the patient’s specific condition. ● Minimal sedatives: Dexmedetomidine was used for sedation at a maintenance dose of 0.2–1.5 μg/(kg·h). The RASS was evaluated every 30 min or 1 h to adjust the drug dose in time. The RASS was maintained at -2 to 0. ● Maximal humane care: Increasing the frequency and length of family visits, increasing clock configurations, and utilizing non-verbal communication skills. 2/ Early rehabilitation: When the RASS is >-3, the patients started early activity. Early activities follow the principle of gradual, stepwise progression. Early rehabilitation treatment consists of four stages: ● Turning over and patting twice a day; active and passive joint exercises; grip strength training using grip ball, four times a day for 30 minutes. ● Incremental semi-fowler. ● Standing besides bed or sitting in chair, once or twice a day for 30 minutes. ● Walk was prescribed once a day for 30 minutes	Incidence of ICU-AW: The study intervention led to a significant decrease in the ICU-AW incidence from 23% to 7% (*P* < 0.001). MRC-Score: The control group (45.28 ± 9.35) was significantly lower than in the intervention group (51.38 ± 8.35) (*P* < 0.001). Incidence of delirium: The control group (n = 11) were more prone to occur delirium than the intervention group (n = 3) (*P* < 0.05). Duration of mechanical ventilation: Intervention based on the eCASH concept reduced mechanical ventilation time (*P* < 0.001). Duration of ICU stay: Shorter ICU stays in the intervention group (8.92 ± 5.35) compared to the control group (14.10 ± 5.52) (*P* < 0.001).
Hodgson et al. (2016) Australia	Theory-based: The goal-oriented theory	Active exercise was conducted depended on the patients’ IMS and were as follows: ● I (IMS = 0): Additional sitting for twice per day. ● II (IMS = 1–2): Active bed exercise was prescribed for 30 minutes. ● Ⅲ (IMS = 3): Active exercise (sitting balance and dangling) was prescribed for 30 minutes. ● IV (IMS = 4–6): Active exercise (stand, standing balance, sit to stand) was prescribed for 45 minutes. ● V (IMS = 7–10): Walk was prescribed for 1 hour.	Incidence of ICU-AW: The incidence of ICU-AW was 50% in the control group compared to 28% in the intervention group, but no statistical significant found (*P* = 0.13). MRC-Score: The control group (45.2 ± 13.2) was lower than in the intervention group (50.4 ± 7.5), but no statistical significant found (*P =* 0.1). The score of IMS: the mean IMS was 7.5 (95% CI: 6.5–8.5) for intervention group and 5.6 (95% CI: 4.6–6.6) for control group (*P* < 0.05). The highest level of activity and duration of active exercise: The results of the intervention group were significantly better than the control group (*P* < 0.001).
Lin et al. (2023) China	Theory-based: The goal-oriented theory	The rehabilitation program was performed according to the patients’ IMS, with a corresponding activity type, duration, frequency, and intensity. Patient’s specific interventions are as follows: ● I (IMS = 0): NMES (twice a day) for 30 minutes; passive motion for limbs (three times a day) for 20 minutes. ● II (IMS = 1–2): Respiratory training/active motion for limbs for 15 minutes; semi-fowler (30–45°) for 20 minutes (twice a day). ● Ⅲ (IMS = 3): Grip strength training for 5 minutes; semi-fowler (45–60°) for 10 minutes; in-bed cycling for 15 minutes; active bedside sitting (twice a day) for 20 minutes. ● IV (IMS = 4–6): Standing balance for 5 minutes; sitting in chair (three times a day) for 20 minutes; in-bed cycling (twice a day) for 20 minutes. ● V (IMS = 7–10): Ambulation training (twice a day) for 10 minutes.	**T1 (discharged from the ICU):** Incidence of ICU-AW, MRC-Score and grip strength: The intervention group was all better than the control group (*P* < 0.05). **T2 (discharged from the hospital):** Incidence of ICU-AW, MRC-Score and grip strength: The intervention group was all better than the control group (*P* < 0.05). Duration of mechanical ventilation: Intervention based on the goal-oriented theory reduced mechanical ventilation time (*P* < 0.05). Length of ICU stay and total hospital stay: The intervention group were shorter than the control group in length of ICU stay and total hospital stay (*P* < 0.05). BI score: The intervention group scored (87.82 ± 10.93) significantly higher than the control group (80.79 ± 13.13) (*P* < 0.05).
Yu et al. (2021) China	Theory-based: The 4E model	1/ Participation: Form a core group of multidisciplinary teams; help patients understand the importance of early rehabilitation; identify barriers to implementation; and develop a training process and program based on clinical practice and evidence. 2/ Education: Medical staff mastered the knowledge and skills of early rehabilitation through theoretical lectures and operation demonstrations. 3/ Implementation: Evaluate patients to analyse the need for early rehabilitation; determine training levels; and conduct dynamic assessments. Patient’s specific interventions are as follows: ● I (RASS ≤ -2): Passive motion for limbs; turn over and pat every 2 hours; and Lower Extremity Pneumatic Therapeutic Apparatus (twice a day). ● II (-1 ≤ RASS ≤ 1): Fist clenching, arm raising, ankle-pumping for 20 sets (twice a day); in-bed cycling for 10 minutes (twice a day); incremental semi-fowler (30°-60°-90°) for 30 minutes (twice a day). ● Ⅲ (Consciousness, muscle strength of both upper limbs at grade 4 or above): The contents of II and active bedside sitting (twice a day) for 30 minutes. 4/ Assessment: Analyze and solve problems in the implementation of the program.	Incidence of ICU-AW: The incidence of ICU-AW was 4% in the intervention group and 13% in the control group (*P* < 0.05). MRC-Score: The control group (45.42 ± 9.81) was significantly lower than in the intervention group (53.60 ± 8.63) (*P* < 0.05). Incidence of delirium and duration of delirium: The incidence of delirium and duration of delirium were lower in the intervention group than the control group (*P* < 0.05). Duration of mechanical ventilation: The duration of mechanical ventilation in the control group (277.49 ± 144.42) was longer than the intervention group (205.50 ± 105.62) (*P* < 0.05). Duration of ICU stay and total hospital stay: Shorter ICU stays (13.47 ± 4.91) and total hospital stays (23.97 ± 7.50) in the intervention group compared to the control group (ICU stays: 16.72 ± 6.75; total hospital stays: 28.03 ± 9.49) (*P* < 0.05).
Zhang et al. (2023) China	Theory-based: The protection motivation theory	1/ Early rehabilitation: ● Complete compensation system (BI > 60): Active motion (including upper limb rotation, lifting movements, hip joint, knee flexion exercise etc.) for 20–30 minutes (once a day), and appropriate resistance exercise (3 kg elastic band for upper limb resistance training, 10 repetitions per set, 4 sets per day). Sit and stand for 20–30 minutes and twice a day. Partial compensation system (40 ≤ BI < 60): Passive motion (including wrist, elbow, shoulder, hip, knee and ankle joints, and the activity was performed once a day for 15–20 minutes) and active motion (maintaining a sitting position for 20–30 minutes and twice a day). ● Supporting education system (BI < 40): Passive motion (passive motion of the fingers, wrist and shoulder joints in each direction were repeated 10 times). 2/ Nutritional support: ● Nutritional risk screening and nutritional assessment was carried out (NRS 2002 and PG-SGA). ● Patients with NRS 2002 ≥ 3 points were given an oral dietary intake if they could take it orally, and early continuous EN within 48 hours if they could not take it orally. Patients with severe malnutrition (Score C of PG-SGA), high nutritional risk (NRS 2002 ≥ 5), and contraindications to EN will receive early progressive low-dose PN within 3–7 days. ● Gastric residual volume, blood glucose, and blood electrolytes were monitored during nutritional support.	Incidence of ICU-AW: ICU-AW occurred more in the control group (18/60) compared to the intervention group (7/60) (*P* < 0.05). Daily activities: BI scores of patients in the control group were lower than the intervention group (*P* < 0.001). Nutritional status: Statistical significance was found in the PG-SGA (*P* < 0.05), total protein and albumin (*P* < 0.001) between the two groups. Duration of mechanical ventilation: The study intervention reduced the duration of mechanical ventilation (*P* < 0.001). Duration of ICU stay and total hospital stay: The intervention led to a reduction in ICU stay (*P* < 0.001) and a total hospital stay (*P* < 0.001).
Frade-Mera et al. (2022) Spain	Evidence-based: ABCDE bundle implementation	The study consisted of three main intervention programs, which were protocol with analgosedation algorithms (“ABC”-including item 1, 2 and 3), delirium prevention and management protocol (“D”-only including item 4) and early mobilization protocol (“E”-only including item 5). 1/ Assess, prevent, and manage pain. 2/ Sedation and agitation assessment with a validated scale, SAT and SBT. 3/ Choice of analgesia and sedation (considering drug metabolism, dose, titration, and discontinuation). 4/ Delirium: Assess, prevent, and manage. 5/ Early mobility and exercise.	MRC-Score: The intervention of “ABC” and “E” didn’t improve MRC-score. The intervention of “D” could be effective in improving MRC-score (*P* < 0.05). Level of mobility: The "E" intervention resulted in more active days for patients with an IMS score of 0 to 2 (*P* < 0.001). Pain assessment: The patient’s pain could be effectively reduced by the “D” intervention (*P* < 0.001) and the "E" intervention (*P* < 0.05).
Zhao et al. (2022) China	Evidence-based: ABCDE bundle implementation	Early multidisciplinary collaboration combined with cluster strategy: 1/ AB-Spontaneous awakening and breathing coordination intervention: SAT and SBT were performed by a nurse and a respiratory therapist at 09:00 and 16:00 every day for the patients. 2/ C-Selection of sedative drugs: The nurse observes the patient’s pain changes, adjusts the speed of the drug in time, and gives feedback to the doctor. 3/ D-False evaluation and management: The mental status was assessed by RASS (RASS was assessed at 09:00 every day). If the assessment score was >-3, the assessment was performed by a psychiatrist using the CAM-ICU. 4/ E-Early rehabilitation: The individualized rehabilitation training was performed by rehabilitation physician, cardiologist and nurse. Rehabilitation treatment consists of five stages according to extremities muscle strength: main and passive activity training for limbs and chest; increase turnaround movement; ensure the stability of vital signs; increase standing activity beside bed; increase underground activity.	MRC-Score: Lower MRC-Score in the control group (41.7 ± 2.05) compared to the intervention group (53.2 ± 1.28) (*P* < 0.001). Duration of mechanical ventilation: The duration of mechanical ventilation was significantly higher in the control group (8.6 ± 3.47) than the intervention group (5.5 ± 2.51) (*P* < 0.001). BI score: The BI score was 63.2 ± 6.88 in the control group compared to 83.6 ± 7.14 in the intervention group (*P* < 0.001). Complications: Pressure sores, pneumonia and deep venous thrombosis were more likely to occur in the control group (*P* < 0.05). SF-36: There were statistically significant differences between these two groups in all eight dimensions of the SF-36 (*P* < 0.001).
Ying W et al. (2021) China	Evidence-based: Bundle implementation	The main interventions included airway management and breathing coordination intervention, analgesic and sedation management, prevention of complications (stress ulcer, thromboembolic prophylaxis) and early rehabilitation. Early rehabilitation interventions were as follows: ● I (Unconscious): Turn over every 2 hours; passive motion (three times a day) for 15–20 minutes. ● II (Consciousness): Semi-fowler (30–45°) for 20 minutes and active resistance training (three times a day); turn over every 2 hours. ● Ⅲ (Muscle strength of both upper limbs at grade 3 or above): Active bedside sitting and active resistance training for 15–20 times, and the contents of II. ● IV (Muscle strength of both lower limbs at grade 3 or above): Standing besides bed or sitting in chair, in-bed cycling for 20 minutes; the contents of Ⅲ.	Incidence of ICU-AW: The bundle led a decline in ICU-AW incidence (*P* < 0.05). Incidence of delirium: The incidence of delirium was 13% in the control group compared to 2% in the intervention group (*P* < 0.05). Complications: Ventilator-associated pneumonia was more common in control group (*P* < 0.05). Duration of mechanical ventilation: The bundle implementation led a reduction in length of mechanical ventilation (*P* < 0.001). Duration of ICU stay: The duration of ICU stay was 13.06 ± 3.63 in the control group compared to 9.03 ± 2.43 in the intervention group (*P* < 0.001).
Han et al. (2020) China	Evidence-based: Intervention programme after literature search and expert consultation	The nursing intervention consists of 4 parts: early rehabilitation, psychological care, nutritional support and effectiveness evaluation. 1/ Early rehabilitation: I (RASS ≤ -2 or/and muscle force < 3): NMES and passive motion (twice a day) for 30–60 minutes. ● II (-1 ≤ RASS ≤ 1, muscle force = 3): Sitting on the bed and anti-gravity exercise for 30–60 minutes (twice a day). ● Ⅲ (-1 ≤ RASS ≤ 1, muscle force of both upper limbs at grade 3 or above): Bedside sitting and active resistance training for 30–60 times (twice a day); and the contents of II. ● IV (-1 ≤ RASS ≤ 1, muscle force of both lower limbs at grade 3 or above): Sitting in chair for 30–60 minutes (twice a day); and the contents of Ⅲ. 2/ Psychological care: Psychological care by medical staff and family members. 3/ Nutritional support: ● Nutritional risk screening using NRS 2002. ● Patients with NRS 2002 scores less than 3 do not require nutritional support. ● Patients with NRS 2002 ≥ 3 points require nutritional support. Patients unable to eat by mouth were given early EN within 48h; PN was initiated within 3–7 days in patients who were unable to feed by mouth and have contraindications to EN. 4/ Effectiveness evaluation: Safety and effectiveness evaluation.	Incidence of ICU-AW: The incidence of ICU-AW was 5% in the intervention group and 18% in the control group (*P* < 0.05). MRC-Score: The MRC-score in the intervention group (51.51 ± 6.42) significantly higher than that in the control group (44.34 ± 6.97) (*P* < 0.001). BI score: The BI score was 50.29 ± 19.81 in the control group compared to 26.14 ± 19.22 in the intervention group (*P* < 0.001). Duration of ICU stay: Longer ICU stay in the control group compared to the intervention group (*P* < 0.05).

Abbreviations: RASS, the Richmond Agitation-Sedation Scale; MRC, Medical Research Council Muscle Score; IMS, ICU Mobility Scale score; BI, Barthel Index; NMES, Neuromuscular Electrical Stimulation; NRS 2002, Nutritional Risk Screening 2002; PG-SGA, Patient-Generated Subjective Global Assessment; EN, Enteral Nutrition; PN, Parenteral Nutrition; SAT, Spontaneous Awakening Trials; SBT, Spontaneous Breathing Trials; CAM-ICU, the Confusion Assessment Method for The Intensive Care Unit; SF-36, the MOS item short from health survey.

### Data analysis and synthesis

Differences in clinical outcomes and methodological approaches precluded the meta-analyses conducted in this study. The data were therefore analysed narratively and synthesised using the SWiM guidelines [17]. After data extraction, the data were synthesised and discussed by the research team.

## Results

### Search results and study descriptions

**Fig 1:**
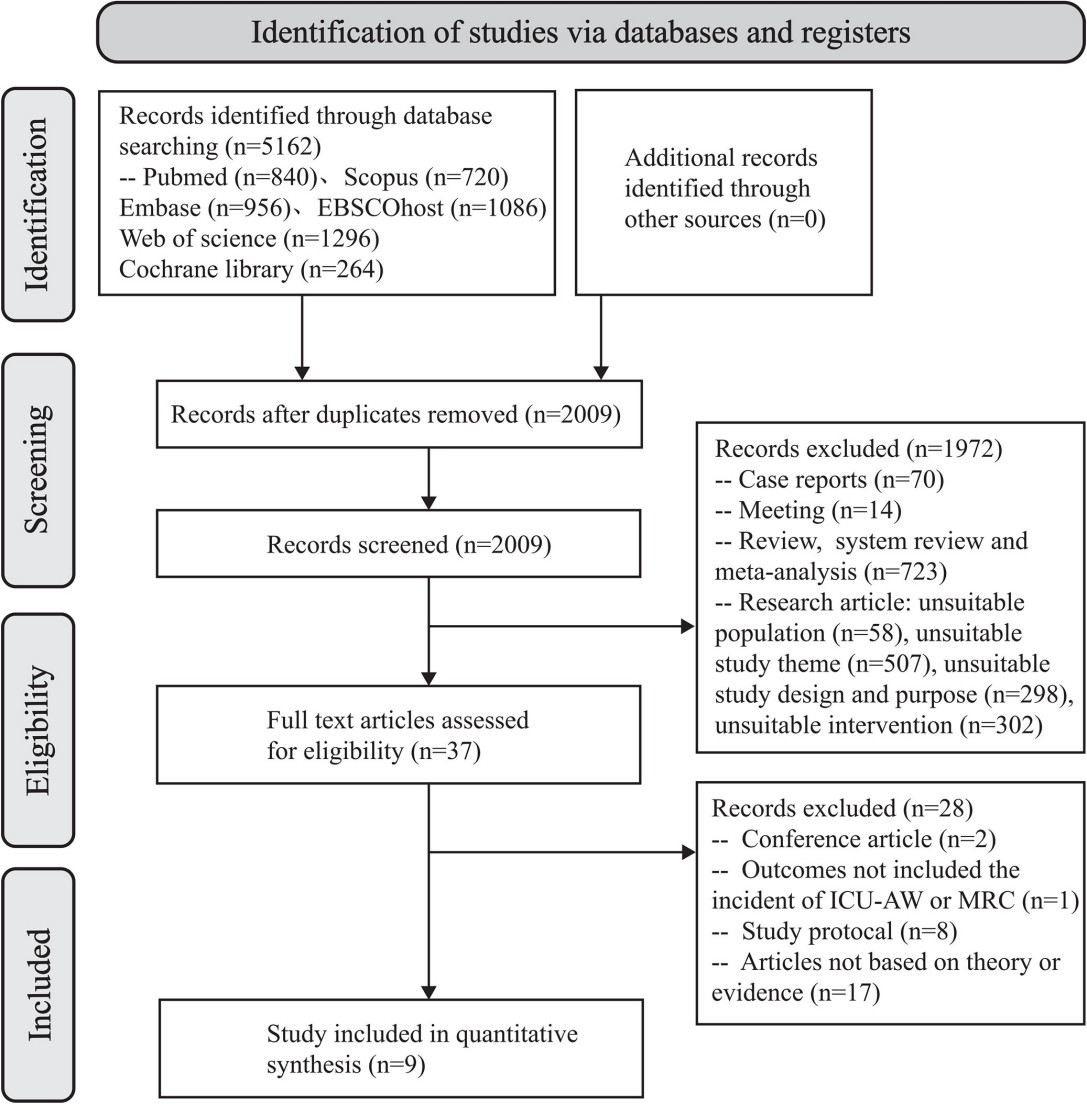
Prisma flow diagram.

A total of 5162 studies were retrieved, 3153 of which were duplicates. The full texts of 37 studies were scrutinised by researchers, and 28 documents were excluded for various reasons. Ultimately, 9 studies were included in the final analysis. A PRISMA statement flow chart was used throughout the study (Fig 1).

In total, nine studies were included in this review, of which five were RCTs [15, 18–21], one was an observational cohort study [22] and three were quasi-experimental studies [23–25]. Seven studies were conducted in China [18–21, 23–25], one in Spain [22], and one in Australia [15].

### Methodological appraisal

The inter-rater agreement between the two researchers in the quality appraisal was 96.12% (Kappa: 0.89). Regarding the quality of included studies, two RCTs [15, 18] were high and three RCTs [19–21] were moderate. Three quasi-experimental studies [23–25] and one cohort study [22] were all considered high. The quality assessment of the included literature can be found in Supporting information.

### Description of the population

A total of 1,391 participants from 9 studies participated in this study (the number of participants included from each study ranged from 50 to 605). The participants were all older than 18 years, and the patient age groups ranged between 40 and 70 years. In terms of sex differences, there were significantly more males than females in the included studies.

The participants included in this study were all critically ill patients in the ICU. However, patients are admitted to the ICU for different reasons. Most of the studies were performed in a single ICU, whereas three studies were conducted in multiple ICUs [15, 22, 25]. Three studies did not specify the diseases of the included patients [15, 21, 23]. Four studies [20, 22, 24, 25] included patients with a wide range of diseases, including respiratory, cardiovascular, neurological, and endocrine diseases. Two studies included patients with a single disease, namely, myocardial infarction in the study by Zhao et al. [19] and acute type A aortic coarctation in the study by Lin et al. [18]. None of the included studies were of low methodological quality (specific details can be found in Supporting information). The characteristics of the included studies are detailed in Table 1, and the interventions and outcomes of the included studies are detailed in Table 2.

### Descriptions of interventions

#### Contents of the nursing interventions

Preventive nursing interventions for ICU-AW mainly included physiotherapy, nutritional support, airway management, sedation and analgesia management, complication prevention (delirium, stress injury and deep vein thrombosis prevention), and psychological care.

Nursing interventions in three studies were all based on physiotherapy [15, 18, 25]. Most studies utilised a combination of multiple nursing interventions. One study combined sedation and analgesia management, physiotherapy and psychological care [24]. One study combined physiotherapy with nutritional support [23]. In addition, one study added psychological care to physiotherapy and nutritional support [20]. Nursing interventions in three studies focused on physiotherapy, airway management, sedation and analgesia management, and prevention of complications [19, 21, 22].

To assess the timing of early rehabilitation, two studies [15, 18] used the ICU Mobility Scale score as a criterion, one study [23] used the Barthel Index score, one study [19] used muscle strength, and three studies [20, 21, 25] used awareness and muscle strength.

Only two studies addressed the timing of nutritional support, with one [23] using both the Nutritional Risk Screening 2002 and Patient-Generated Subjective Global Assessment as the criteria and the other [20] using only the Nutritional Risk Screening 2002 as the criterion.

#### Application of theory and evidence to nursing intervention

The literature included was categorised into theory-based and evidence-based nursing interventions based on the content of the intervention. One study’s nursing intervention was based on eCASH [24], two studies were based on goal-oriented theory [15, 18], one study was based on the 4E model [25], and another study was based on protection motivation theory with Orem theory [23]. In this last study, the evidence-based nursing intervention was primarily an "ABCDE" bundle [19, 21, 22].

### Descriptions of control interventions

The intervention groups of all the studies were compared with a control group. Most of the studies described the interventions for the control group [15, 18, 19, 21, 23–25]. The control group mainly received conventional nursing interventions, which included (1) monitoring vital signs, (2) keeping the patient’s limbs in the functional position, (3) turning over and patting the back every 2 h, (4) performing functional exercises for the limbs, and (5) elevating the head of the bed. All usual practices were continued, with no restrictions on physical therapy or sedation.

### Main results on outcomes

The included studies reported the incidence of ICU-AW or total MRC score in critically ill patients in the ICU. Only one study reported no statistically significant difference in ICU-AW incidence or MRC scores between the intervention and control groups, but nursing interventions based on goal-directed theory reduced the incidence of ICU-AW and improved MRC scores in critically ill patients [15]. In other studies, theory-based or evidence-based interventions were effective in reducing the incidence of ICU-AW and improving the MRC score in critically ill patients in the ICU.

Three studies reported data on delirium, mechanical ventilation, and ICU stays [21, 24, 25]. In addition, three other studies reported ICU stays [20, 18, 23], and another three studies reported mechanical ventilation [19, 18, 23]. Two studies described complications [19, 21], three studies reported BI scores [19, 20, 18], and three studies assessed mobility [15, 22, 23]. Furthermore, nutritional status [23], pain assessment [22], and SF-36 measurement [19] were mentioned in separate studies. The results showed that theory-based or evidence-based nursing interventions were effective in improving these indicators.

## Discussion

### Specific nursing interventions

This review included 1391 participants from 9 studies. Although the interventions in the included studies were physiotherapy (NMES and early rehabilitation) and nutritional support, their specific interventions were different (e.g., timing, frequency, and content of early rehabilitation). The outcomes of the interventions remained similar.

The contents and forms of early rehabilitation were diverse and include functional exercises for the limbs, joint mobility training, custom-made rehabilitation bed chairs [26], and in-bed cycling/stepping [27]. In this study, early rehabilitation was the most important measure to prevent ICU-AW in critically ill patients. Early rehabilitation in the included literature was mainly based on passive bed training, passive bed training and active resistance exercise, bedside sitting and standing activities, and walking and joint mobility training. Notably, the timing of early rehabilitation of critically ill patients was significant, and the commonly used indicators were consciousness, muscle strength, and mobility (such as the ICU Mobility Scale score and Barthel Index). In previous meta-analyses [28, 29], early rehabilitation units were found to be effective at preventing ICU-AW and reducing ICU and hospital stays. This finding is also consistent with our findings. Therefore, this emphasises the key role and position of early rehabilitation in the prevention of ICU-AW. Moreover, virtual reality (VR) has been notably used in early rehabilitation in the ICU [30]. Gomes et al. used the Nintendo Wii™ with 60 adult ICU patients to increase their physical activity and promote recovery [31]. The study showed that the use of VR could also reduce anxiety and depression levels in patients and reduce the incidence of post-traumatic stress disorder [32, 33]. In addition, a study by Ryo et al. revealed that the use of VR reduced the dose of fentanyl administered and relieved respiratory depression [34]. The value of the use of VR with early rehabilitation in the ICU is worth exploring.

In terms of nutrition, early implementation of standardised enteral nutrition in critically ill patients reduces the prevalence of ICU-AW and prevents acute muscle loss [12, 35]. The decision to provide nutritional support to critically ill patients generally depends on the patient’s own nutritional status, which is generally assessed by the Nutritional Risk Screening 2002. In patients requiring nutritional support, high protein delivery may be more beneficial to critically ill patients than medium or low protein delivery [36, 37]. Early parenteral nutrition also needs to be avoided. Late parenteral nutrition reduces the incidence of ICU-AW and may accelerate patient recovery compared with early parenteral nutrition [38]. In the included studies, the researchers emphasised that early enteral nutrition is essential for critically ill patients [20, 23]. Additionally, continuous monitoring of patients’ gastric residual volumes and vital signs is required during enteral nutrition.

Recently, many studies have increasingly focused on the effects of multiple interventions rather than a single intervention to prevent ICU-AW in critically ill patients. Verceles et al. intervened in critically ill patients through exercise, protein supplementation and electrical stimulation [39]. In the study by Zhou et al., the intervention focused on early rehabilitation and early nutrition [40]. Multiple intervention methods are becoming increasingly popular. In this review, the included studies favoured a combination of multiple nursing interventions. The American Association of Critical Care Nursing proposed the ABCDE bundle based on an evidence-based medical foundation. The ABCDE bundle is a collection of multicomponent interventions that are highly effective in preventing ICU-AW in critically ill patients. The ABCDE bundle includes Assess, Prevent, and Manage Pain (A), Spontaneous Awakening Trials (SAT) and Spontaneous Breathing Trials (SBT) (B), Choice of analgesia and sedation (C), Delirium: Assess, Prevent, and Manage (D), and Early mobility and Exercise (E). Researchers have focused their attention on airway management, sedation and analgesia management, prevention of complications, and psychological care for both the ABCDE bundle and other existing evidence-based interventions [22, 41].

In addition, glycaemic control and Chinese medicine care have also been used in the management of ICU-AW.

Hyperglycaemia, as part of the endocrine metabolic response after stress, is present in almost all critically ill patients and is associated with a poor prognosis. Intensive insulin therapy may reduce morbidity and mortality by preventing vital organ dysfunction and new severe infections [42]. Hyperglycaemia is also a high-risk factor in ICU-AW [43], making glucose monitoring and glycaemic control in critically ill patients very important.

The application of TCM in ICU-AW has been a hot topic in recent years. Wei et al. reported that "hybrid treatment" of mechanically ventilated ICU patients with various Chinese medicine methods—such as modifying the components of Buzhong Yiqi Decoction, along with press needles and ear point buried beans—significantly improved the clinical symptoms of ICU-AW, shortened the duration of mechanical ventilation, and improved patient quality of life [44]. In addition, Wang et al. reported that early electrical stimulation of acupoints (Huantiao-GB 30, Futu-ST 32, Zusanli-ST 36, Xuanzhong-GB 39 and Taichong-LR 3) improved lower limb muscles in ICU-AW patients due to septic shock [45]. Studies in recent years have shown that TCM has a role in improving muscle atrophy in patients, which also provides ideas and inspiration for the future care of ICU-AW patients [46].

In conclusion, the content of nursing interventions in ICU-AW is unlikely to be a single intervention; instead, it is likely to focus on holistic, integrated, and individualised interventions.

### Application of theory and evidence

Considering the high morbidity and mortality of ICU-AW, nurses can play an important role in the management of ICU-AW by providing theory-based and evidence-based interventions. This systematic review revealed that the application of nursing theory in the ICU-AW mainly included the eCASH concept, the goal-oriented theory, the 4E model and the protection motivation theory. The main evidence-based nursing intervention for ICU-AW is the ABCDE bundle.

Designed to establish optimal patient comfort with minimal sedation, eCASH is widely used in ICU patients [47]. In addition, "B" in the content of the "ABCDE" bundle refers to sedation and analgesia interventions. Li et al. analysed the risk factors for ICU-AW in mechanically ventilated patients and reported that the duration of sedative medication is one of the high-risk factors for ICU-AW [48]. This coincides with the eCASH and ABCDE bundle, which reduces the use of sedation and maintains minimal sedation through the use of spontaneous awakening trials or targeted sedation strategies (continuous titration of sedation to maintain minimal sedation).

Nursing interventions based on goal-directed theory enable nurses to clarify relevant nursing measures through clear mini-goals, ultimately leading to precise care for patients. The studies of Hodgson [15] et al. and Lin [18] et al. used goal-directed theory to guide early rehabilitation of critically ill patients, thereby preventing ICU-AW with delirium. A study by Schaller et al. reported the same results [49], with early and goal-directed mobilisation improving patient activity, shortening the length of stay in the SICU, and improving functional mobility at hospital discharge. Goal-directed theory can be applied to the nutritional support of patients in addition to early rehabilitation.

The 4E model and the protective motivation theory have also been used in critically ill patients. Moreover, transition and empowerment theory [50–52] and care theory [53–55] have been widely applied to the care of ICU patients.

In conclusion, comprehensive interventions based on theory or evidence are needed to guide clinical practice and promote patient recovery in the future.

### Limitations

This systematic review has certain limitations and shortcomings; however, these limitations cannot be avoided. First, the interventions and study designs of the studies included in this review were heterogeneous, which meant that a meta-analysis was not possible. Second, the number of included studies was small, with most of the studies conducted in China; thus, language bias must be assumed. Finally, publication bias cannot be ruled out. Therefore, high-quality and large RCTs are needed to strengthen the credibility and generalisation of the findings.

## Conclusions

In this systematic review, theory- and evidence-based interventions reduced the incidence of ICU-AW (or improved MRC scores) and reduced the incidence of delirium and the hospital length of stay in all included studies. This review provides guidelines on the use of early rehabilitation, nutritional support, airway management, sedation and analgesia management, complication prevention, and psychological care in preventing ICU-AW. We also discuss other nursing interventions that may be applied to ICU-AW. Future studies investigating the impact of theory- and evidence-based nursing interventions on the prevention of ICU-AW in critically ill patients should integrate different interventions. In addition, further studies are needed to investigate theory- and evidence-based nursing interventions for critically ill patients.

## Supporting information

S1 Checklist PRISMA 2020 checklist for the present systematic review. (DOCX)

S1 File Search strategies. (DOCX)

S1 Table JBI critical appraisal checklist for randomized controlled trials. (DOCX)

S2 Table JBI critical appraisal checklist for quasi-experimental studies. (DOCX)

S3 Table JBI critical appraisal checklist for cohort study. (DOCX)
