# Supplementary material for: Theory-based and evidence-based nursing interventions for the prevention of ICU-acquired weakness in the intensive care unit: A systematic review
Source: PLoS One. 2024 Sep 13;19(9):e0308291. doi: 10.1371/journal.pone.0308291 (PMC11398680; doi:10.1371/journal.pone.0308291)
Supplement: S1 File — (DOCX) [file pone.0308291.s002.docx]

**Search strategies**

1. **PUBMED SEARCH STRATEGY (n=840)**

| **PUBMED SEARCH STRATEGY** | | | |
| --- | --- | --- | --- |
| Search number | Query | Search Details | Results |
| 3 | (((((Intensive care unit acquired weakness) OR (ICU-AW)) OR (ICUAW)) OR (ICU-acquired weakness)) OR (Medical Research Council Muscle Score (MRC))) AND ((((((((intensive care) OR (intensive care unit)) OR (intensive care units)) OR (ICU)) OR (critically ill patients)) OR (Critical Illness)) OR (critically ill)) OR (Critical care)) | ((("intensive care units"[MeSH Terms] OR ("intensive"[All Fields] AND "care"[All Fields] AND "units"[All Fields]) OR "intensive care units"[All Fields] OR ("intensive"[All Fields] AND "care"[All Fields] AND "unit"[All Fields]) OR "intensive care unit"[All Fields]) AND ("acquirable"[All Fields] OR "acquire"[All Fields] OR "acquired"[All Fields] OR "acquirement"[All Fields] OR "acquirements"[All Fields] OR "acquires"[All Fields] OR "acquiring"[All Fields]) AND ("frailty"[MeSH Terms] OR "frailty"[All Fields] OR "weakness"[All Fields] OR "weaknesses"[All Fields])) OR "ICU-AW"[All Fields] OR "ICUAW"[All Fields] OR ("ICU-acquired"[All Fields] AND ("frailty"[MeSH Terms] OR "frailty"[All Fields] OR "weakness"[All Fields] OR "weaknesses"[All Fields])) OR ((("biomedical research"[MeSH Terms] OR ("biomedical"[All Fields] AND "research"[All Fields]) OR "biomedical research"[All Fields] OR ("medical"[All Fields] AND "research"[All Fields]) OR "medical research"[All Fields]) AND ("council"[All Fields] OR "council s"[All Fields] OR "councils"[All Fields]) AND ("muscle s"[All Fields] OR "muscles"[MeSH Terms] OR "muscles"[All Fields] OR "muscle"[All Fields]) AND ("score"[All Fields] OR "score s"[All Fields] OR "scored"[All Fields] OR "scores"[All Fields] OR "scoring"[All Fields] OR "scorings"[All Fields])) AND ("magn reson chem"[Journal] OR "mrc"[All Fields]))) AND ("critical care"[MeSH Terms] OR ("critical"[All Fields] AND "care"[All Fields]) OR "critical care"[All Fields] OR ("intensive"[All Fields] AND "care"[All Fields]) OR "intensive care"[All Fields] OR ("intensive care units"[MeSH Terms] OR ("intensive"[All Fields] AND "care"[All Fields] AND "units"[All Fields]) OR "intensive care units"[All Fields] OR ("intensive"[All Fields] AND "care"[All Fields] AND "unit"[All Fields]) OR "intensive care unit"[All Fields]) OR ("intensive care units"[MeSH Terms] OR ("intensive"[All Fields] AND "care"[All Fields] AND "units"[All Fields]) OR "intensive care units"[All Fields]) OR ("intensive care units"[MeSH Terms] OR ("intensive"[All Fields] AND "care"[All Fields] AND "units"[All Fields]) OR "intensive care units"[All Fields] OR "icu"[All Fields]) OR (("critical illness"[MeSH Terms] OR ("critical"[All Fields] AND "illness"[All Fields]) OR "critical illness"[All Fields] OR ("critically"[All Fields] AND "ill"[All Fields]) OR "critically ill"[All Fields]) AND ("patient s"[All Fields] OR "patients"[MeSH Terms] OR "patients"[All Fields] OR "patient"[All Fields] OR "patients s"[All Fields])) OR ("critical illness"[MeSH Terms] OR ("critical"[All Fields] AND "illness"[All Fields]) OR "critical illness"[All Fields]) OR ("critical illness"[MeSH Terms] OR ("critical"[All Fields] AND "illness"[All Fields]) OR "critical illness"[All Fields] OR ("critically"[All Fields] AND "ill"[All Fields]) OR "critically ill"[All Fields]) OR ("critical care"[MeSH Terms] OR ("critical"[All Fields] AND "care"[All Fields]) OR "critical care"[All Fields])) | **840** |
| 2 | (((((((intensive care) OR (intensive care unit)) OR (intensive care units)) OR (ICU)) OR (critically ill patients)) OR (Critical Illness)) OR (critically ill)) OR (Critical care) | "critical care"[MeSH Terms] OR ("critical"[All Fields] AND "care"[All Fields]) OR "critical care"[All Fields] OR ("intensive"[All Fields] AND "care"[All Fields]) OR "intensive care"[All Fields] OR ("intensive care units"[MeSH Terms] OR ("intensive"[All Fields] AND "care"[All Fields] AND "units"[All Fields]) OR "intensive care units"[All Fields] OR ("intensive"[All Fields] AND "care"[All Fields] AND "unit"[All Fields]) OR "intensive care unit"[All Fields]) OR ("intensive care units"[MeSH Terms] OR ("intensive"[All Fields] AND "care"[All Fields] AND "units"[All Fields]) OR "intensive care units"[All Fields]) OR ("intensive care units"[MeSH Terms] OR ("intensive"[All Fields] AND "care"[All Fields] AND "units"[All Fields]) OR "intensive care units"[All Fields] OR "icu"[All Fields]) OR (("critical illness"[MeSH Terms] OR ("critical"[All Fields] AND "illness"[All Fields]) OR "critical illness"[All Fields] OR ("critically"[All Fields] AND "ill"[All Fields]) OR "critically ill"[All Fields]) AND ("patient s"[All Fields] OR "patients"[MeSH Terms] OR "patients"[All Fields] OR "patient"[All Fields] OR "patients s"[All Fields])) OR ("critical illness"[MeSH Terms] OR ("critical"[All Fields] AND "illness"[All Fields]) OR "critical illness"[All Fields]) OR ("critical illness"[MeSH Terms] OR ("critical"[All Fields] AND "illness"[All Fields]) OR "critical illness"[All Fields] OR ("critically"[All Fields] AND "ill"[All Fields]) OR "critically ill"[All Fields]) OR ("critical care"[MeSH Terms] OR ("critical"[All Fields] AND "care"[All Fields]) OR "critical care"[All Fields]) | 715, 816 |
| 1 | ((((Intensive care unit acquired weakness) OR (ICU-AW)) OR (ICUAW)) OR (ICU-acquired weakness)) OR (Medical Research Council Muscle Score (MRC)) | (("intensive care units"[MeSH Terms] OR ("intensive"[All Fields] AND "care"[All Fields] AND "units"[All Fields]) OR "intensive care units"[All Fields] OR ("intensive"[All Fields] AND "care"[All Fields] AND "unit"[All Fields]) OR "intensive care unit"[All Fields]) AND ("acquirable"[All Fields] OR "acquire"[All Fields] OR "acquired"[All Fields] OR "acquirement"[All Fields] OR "acquirements"[All Fields] OR "acquires"[All Fields] OR "acquiring"[All Fields]) AND ("frailty"[MeSH Terms] OR "frailty"[All Fields] OR "weakness"[All Fields] OR "weaknesses"[All Fields])) OR "ICU-AW"[All Fields] OR "ICUAW"[All Fields] OR ("ICU-acquired"[All Fields] AND ("frailty"[MeSH Terms] OR "frailty"[All Fields] OR "weakness"[All Fields] OR "weaknesses"[All Fields])) OR ((("biomedical research"[MeSH Terms] OR ("biomedical"[All Fields] AND "research"[All Fields]) OR "biomedical research"[All Fields] OR ("medical"[All Fields] AND "research"[All Fields]) OR "medical research"[All Fields]) AND ("council"[All Fields] OR "council s"[All Fields] OR "councils"[All Fields]) AND ("muscle s"[All Fields] OR "muscles"[MeSH Terms] OR "muscles"[All Fields] OR "muscle"[All Fields]) AND ("score"[All Fields] OR "score s"[All Fields] OR "scored"[All Fields] OR "scores"[All Fields] OR "scoring"[All Fields] OR "scorings"[All Fields])) AND ("magn reson chem"[Journal] OR "mrc"[All Fields])) | 1346 |

1. **EBSCOhost SEARCH STRATEGY (n=1086)**

| **EBSCOhost SEARCH STRATEGY** | | |
| --- | --- | --- |
| # | Search | Results |
| S3 | S1 AND S2 | **1,086** |
| S2 | intensive care OR intensive care unit OR intensive care units[mesh terms] OR icu OR critically ill patients OR critical illness OR critically ill OR critical care | 1,163,290 |
| S1 | intensive care unit acquired weakness OR icuaw OR icu-aw OR icu acquired weakness OR Medical Research Council Muscle Score (MRC) | 1,502 |

EBSCOhost Research Databases include CINAHL Plus with Full Text, MEDLINE and Academic Search Complete.

1. **EMBASE SEARCH STRATEGY (n=956)**

('intensive care unit acquired weakness' OR 'icu-aw' OR 'icuaw' OR 'icu-acquired weakness' OR 'medical research council muscle score (mrc)') AND ('intensive care'/exp OR 'intensive care' OR 'intensive care unit'/exp OR 'intensive care unit' OR 'icu' OR 'critically ill patients' OR 'critical illness'/exp OR 'critical illness' OR 'critically ill'/exp OR 'critically ill' OR 'critical care'/exp OR 'critical care')

1. **SCOPUS SEARCH STRATEGY (n=720)**

( ( TITLE-ABS-KEY ( "intensive care" ) OR TITLE-ABS-KEY ( "intensive care unit" ) OR TITLE-ABS-KEY ( "intensive care units" ) OR TITLE-ABS-KEY ( "ICU" ) OR TITLE-ABS-KEY ( "critically ill patients" ) OR TITLE-ABS-KEY ( "Critical Illness" ) OR TITLE-ABS-KEY ( "critically ill" ) OR TITLE-ABS-KEY ( "Critical care" ) ) ) AND ( ( TITLE-ABS-KEY ( "Intensive care unit acquired weakness" ) OR TITLE-ABS-KEY ( "ICU-AW" ) OR TITLE-ABS-KEY ( "ICUAW" ) OR TITLE-ABS-KEY ( "ICU-acquired weakness" ) OR TITLE-ABS-KEY ( "Medical Research Council Muscle Score (MRC)" ) ) )

1. **WEB OF SCIENCE SEARCH STRATEGY (n=1296)**

| **WEB OF SCIENCE SEARCH STRATEGY** | | |
| --- | --- | --- |
| # | Search | Results |
| #3 | #2 AND #1 | 1,296 |
| #2 | (((((((TS=(intensive care)) OR TS=(intensive care unit)) OR TS=(intensive care units)) OR TS=(ICU)) OR TS=(critically ill patients)) OR TS=(Critical Illness)) OR TS=(critically ill)) OR TS=(Critical care) and Preprint Citation Index (Exclude – Database) | 637,232 |
| #1 | ((((TS=(Intensive care unit acquired weakness)) OR TS=( ICU-AW)) OR TS=( ICUAW)) OR TS=(ICU-acquired weakness)) OR TS=(Medical Research Council Muscle Score (MRC)) and Preprint Citation Index (Exclude – Database) | 1,514 |

1. **COCHRANE LIBRARY SEARCH STRATEGY (n=264)**

| **COCHRANE LIBRARY SEARCH STRATEGY** | | |
| --- | --- | --- |
| # | Search | Results |
| #1 | (Intensive care unit acquired weakness) OR (ICU-AW) OR (ICUAW) OR (ICU-acquired weakness) OR (Medical Research Council Muscle Score (MRC)) (Word variations have been searched) | 818 |
| #2 | (intensive care) OR (intensive care unit) OR (intensive care units) OR (ICU) OR (critically ill patients) (Word variations have been searched) | 76,312 |
| #3 | (Critical Illness) OR (critically ill) OR (Critical care) (Word variations have been searched) | 42,208 |
| #4 | #2 OR #3 | 97,002 |
| #5 | #1 AND #4 | 661 |

Cochrane Reviews-363; Cochrane Protocols Cochrane-32; Trials-264; Editorials-0; Special Collections-0; Clinical Answers-2.
