# Supplementary material for: Theory-based and evidence-based nursing interventions for the prevention of ICU-acquired weakness in the intensive care unit: A systematic review
Source: PLoS One. 2024 Sep 13;19(9):e0308291. doi: 10.1371/journal.pone.0308291 (PMC11398680; doi:10.1371/journal.pone.0308291)
Supplement: S1 Table — (DOCX) [file pone.0308291.s003.docx]

**Quality assessment of the included studies**

S1 Table. JBI Critical Appraisal Checklist for Randomized Controlled Trials

| References | Q1 | Q2 | Q3 | Q4 | Q5 | Q6 | Q7 | Q8 | Q9 | Q10 | Q11 | Q12 | Q13 | Rating |
| --- | --- | --- | --- | --- | --- | --- | --- | --- | --- | --- | --- | --- | --- | --- |
| Hodgson et al. (2016) | Y | Y | Y | UC | N | Y | Y | Y | Y | Y | Y | Y | Y | 11 |
| Lin et al. (2023) | Y | Y | Y | UC | UC | Y | Y | Y | N | Y | Y | Y | Y | 10 |
| Zhao et al. (2022) | Y | UC | Y | UC | UC | UC | Y | Y | N | Y | Y | Y | Y | 8 |
| Wang et al. (2021) | Y | UC | Y | UC | UC | UC | Y | Y | Y | Y | Y | Y | Y | 9 |
| Han et al. (2020) | Y | UC | Y | UC | UC | UC | Y | Y | N | Y | Y | Y | Y | 8 |

Y = yes; N = no; UC = unclear; NA = not applicable; JBI Critical Appraisal Checklist for Randomized Controlled Trials: Q1 = Was true randomization used for assignment of participants to treatment groups?; Q2 = Was allocation to treatment groups concealed?; Q3 = Were treatment groups similar at the baseline?; Q4 = Were participants blind to treatment assignment?; Q5 = Were those delivering treatment blind to treatment assignment?; Q6 = Were outcomes assessors blind to treatment assignment?; Q7 = Were treatment groups treated identically other than the intervention of interest?; Q8 = Was follow up complete and if not, were differences between groups in terms of their follow up adequately described and analyzed?; Q9 = Were participants analyzed in the groups to which they were randomized?; Q10 = Were outcomes measured in the same way for treatment groups?; Q11 = Were outcomes measured in a reliable way?; Q12 = Was appropriate statistical analysis used?; Q13 = Was the trial design appropriate, and any deviations from the standard RCT design (individual randomization, parallel groups) accounted for in the conduct and analysis of the trial?
