# Supplementary material for: Theory-based and evidence-based nursing interventions for the prevention of ICU-acquired weakness in the intensive care unit: A systematic review
Source: PLoS One. 2024 Sep 13;19(9):e0308291. doi: 10.1371/journal.pone.0308291 (PMC11398680; doi:10.1371/journal.pone.0308291)
Supplement: S2 Table — (DOCX) [file pone.0308291.s004.docx]

**Quality assessment of the included studies**

S2 Table. JBI Critical Appraisal Checklist for Quasi-Experimental Studies

| References | Q1 | Q2 | Q3 | Q4 | Q5 | Q6 | Q7 | Q8 | Q9 | Rating |
| --- | --- | --- | --- | --- | --- | --- | --- | --- | --- | --- |
| Bian et al. (2019) | Y | Y | Y | Y | Y | NA | Y | Y | Y | 8 |
| Yu et al. (2021) | Y | Y | Y | Y | Y | NA | Y | Y | Y | 8 |
| Zhang et al. (2023) | Y | Y | Y | Y | Y | NA | Y | Y | Y | 8 |

Y = yes; N = no; UC= unclear; NA = not applicable; JBI Critical Appraisal Checklist for Quasi-Experimental Studies: Q1= Is it clear in the study what is the “cause” and what is the “effect” (i.e. there is no confusion about which variable comes first)?; Q2 = Were the participants included in any comparisons similar?; Q3 = Were the participants included in any comparisons receiving similar treatment/care, other than the exposure or intervention of interest?; Q4 = Was there a control group?; Q5= Were there multiple measurements of the outcome both pre and post the intervention/exposure?; Q6 = Was follow up complete and if not, were differences between groups in terms of their follow up adequately described and analyzed?; Q7 = Were the outcomes of participants included in any comparisons measured in the same way?; Q8 = Were outcomes measured in a reliable way?; Q9 = Was appropriate statistical analysis used?
