# Supplementary material for: Theory-based and evidence-based nursing interventions for the prevention of ICU-acquired weakness in the intensive care unit: A systematic review
Source: PLoS One. 2024 Sep 13;19(9):e0308291. doi: 10.1371/journal.pone.0308291 (PMC11398680; doi:10.1371/journal.pone.0308291)
Supplement: S3 Table — (DOCX) [file pone.0308291.s005.docx]

**Quality assessment of the included studies**

S3 Table. JBI Critical Appraisal Checklist for Cohort study

| References | Q1 | Q2 | Q3 | Q4 | Q5 | Q6 | Q7 | Q8 | Q9 | Q10 | Q11 | Rating |
| --- | --- | --- | --- | --- | --- | --- | --- | --- | --- | --- | --- | --- |
| Mera et al. (2021) | Y | Y | Y | Y | Y | Y | Y | Y | Y | UC | Y | 10 |

Y = yes; N = no; UC = unclear; NA = not applicable; JBI Critical Appraisal Checklist for Cohort study: Q1= Were the two study groups from the same population?; Q2 = Were exposure factors measured in the same way for both groups?; Q3 = Were valid and credible methods used to measure exposure?; Q4 = Were confounding factors identified?; Q5 = Were measures in place to address confounding factors?; Q6 = Was the observed outcome not present in the study population at the start of the study/exposure?; Q7 = Were valid and credible methods used to measure results?; Q8 = Was the duration of follow-up reported, and was the duration of follow-up sufficient to obtain the occurrence of the outcome?; Q9 = Was follow up complete and if not, was the reason for the missed visit described and explored?; Q10 = Were measures taken to address incomplete follow-up visits?; Q11 = Was appropriate statistical analysis used?
